# Review of *Mammarenavirus* Biology and Replication

**DOI:** 10.3389/fmicb.2018.01751

**Published:** 2018-08-03

**Authors:** Steven J. Hallam, Takaaki Koma, Junki Maruyama, Slobodan Paessler

**Affiliations:** ^1^Department of Pathology, University of Texas Medical Branch at Galveston, Galveston, TX, United States; ^2^Department of Microbiology, Tokushima University Graduate School of Medical Science, Tokushima, Japan

**Keywords:** arenavirus, virus replication, lassa virus, junin virus, viral entry, virus immune evasion

## Abstract

The family *Arenaviridae* is divided into three genera: *Mammarenavirus*, *Reptarenavirus*, and *Hartmanivirus*. The *Mammarenaviruses* contain viruses responsible for causing human hemorrhagic fever diseases including New World viruses Junin, Machupo, Guanarito, Sabia, and Chapare virus and Old World viruses Lassa, and Lujo virus. These two groups of arenaviruses share the same genome organization composed of two ambisense RNA segments. These segments contain four open reading frames that encode for four proteins: the nucleoprotein, glycoprotein precursor, L protein, and Z. Despite their genome similarities, these groups exhibit marked differences in their replication life cycles. This includes differences in attachment, entry, and immune evasion. By understanding the intricacy of replication in each of these viral species we can work to develop counter measures against human diseases. This includes the development of vaccines and antivirals for these emerging viral threats. Currently only the vaccine against Junin virus, Candid#1, is in use as well as Ribavirin for treatment of Lassa Fever. In addition, small molecule inhibitors can be developed to target various aspects of the virus life cycle. In these ways an understanding of the arenavirus replication cycle can be used to alleviate the mortality and morbidity of these infections worldwide.

## Family *Arenaviridae*

The family *Arenaviridae* is a viral family encompassing three newly separated genera: *Mammarenavirus*, *Reptarenavirus*, and *Hartmanivirus*. As noted in the names, genus *Mammarenavirus* encompasses those viruses that infect mammalian hosts whereas the reptarenaviruses and hartmaniviruses infect reptilian hosts. The genus *Mammarenavirus* is further sub-divided into the Old World and New World groups. Currently, the International Committee for Taxonomy of Viruses (ICTV) recognizes 41 distinct viral species in the genus *Mammarenavirus* (Maes et al., 2018). All members of the family have a negative sense, bi-segmented genome consisting of a large (L) and small (S) segment. While varying between species on exact length, on average the L segment of arenaviruses is 7.2 kb long whereas the S segment is 3.4 kb (Buchmeier et al., 2007). Each segment contains two open reading frames (ORFs), utilizing an ambisense coding strategy, separated by an intergenic region (IGR). This coding strategy allows for the expression of early and late genes regulated by coding orientation. The L segment encodes the multi-functional matrix protein (Z) and the large RNA-dependent RNA polymerase (RdRp) complex (LP). The S segment encodes the viral nucleoprotein (NP) and glycoprotein precursor complex (GPC) that is later cleaved into a stable signal peptide (SSP), GP1, and GP2. Utilizing only a limited number of proteins, arenaviruses are still able to infect, replicate, and modulate host responses in complex and elegant fashions.

As stated previously, the genus *Mammarenavirus* is separated into two main groups, Old World and New World, based on their genetic, geographic, and epidemiological relationship (Wolff et al., 1978; Charrel et al., 2008). The natural host for Old World arenaviruses is the sub-family *Murinae* of the *Muridae* family of mice. Old World arenavirus species include prototypic members Lymphocytic choriomeningitis virus (LCMV) and Lassa virus (LASV) as well as newly emerged Lujo virus. The Old World viruses are generally geographically confined to the African continent with the exception of LCMV that circulates globally in the *Mus musculus* host. LASV strains were previously subdivided into 4 distinct lineages based on genetic relationship (Bowen et al., 2000; Manning et al., 2015). However, recent phylogenetic evaluation postulates the emergence of a fifth and sixth lineage (Manning et al., 2015; Whitmer et al., 2018). While the first 3 lineages cluster together geographically in Nigeria, lineage 4 LASV strains are found across several countries of West Africa.

The New World arenaviruses are further divided into four clades: A, B, C, and A/Rec (Clade D). The human hemorrhagic pathogens Junin virus (JUNV), Machupo virus (MACV), Guanarito virus (GTOV), Sabia virus (SABV), and Chapare virus, cluster in clade B together with the prototypic Tacaribe virus (TCRV). JUNV, MACV, GTOV, and SABV are the causative agents of Argentine Hemorrhagic Fever (AHF), Bolivian Hemorrhagic Fever (BHF), Venezuelan Hemorrhagic Fever (VHF), and Brazilian Hemorrhagic Fever (BzHF), respectively. Pichinde virus (PICV) is a prototypic clade A virus that is not pathogenic to humans (Buchmeier et al., 1974). To date, it has been widely utilized as a surrogate for LASV pathogenesis, mimicking several disease manifestations in the guinea pig model (Jahrling et al., 1981). Clade A/Rec (Clade D) is hypothesized to be a recombination of clades A and B (Charrel et al., 2001, 2002; Fulhorst et al., 2001; Archer and Rico-Hesse, 2002). This clade includes North American viruses Whitewater Arroyo virus (WWAV) and Bear Canyon virus (BCNV). The New World arenaviruses are geographically located on the South and North American continents. The natural host for the New World viruses is the *Sigmodontinae* sub-family of *Muridae*-family mice (Arenaviridae CDC, 2013), with the exception of TCRV, which is found in *Artibeus* bats (Salazar-Bravo et al., 2002). Recently, TCRV was isolated from *Amblyomma americanum* ticks in Florida opening the possibility of viral evolution aimed at adaptation to arthropod hosts (Sayler et al., 2014). No further studies have been undertaken to quantify the presence of other New World arenaviruses in arthropods.

Recent surveillance has uncovered the circulation of novel arenaviruses in Asia. Wenzhou Virus (WENV) was isolated in China and is phylogenetically related to the Old World viruses (Li et al., 2015). Further studies indicate that a Cambodian strain of this virus can cause human infection (Blasdell, 2016). These viruses have been found in several rodent vectors (Li et al., 2015), leading to the possibility of a large natural host range.

Both Old World and New World viruses cause significant disease burden in their endemic areas of circulation. According to the latest (Centers for Disease Control and Prevention [CDC], 2013, 2015) reports. LASV causes approximately 100,000–300,000 clinical cases of Lassa fever (LF) a year and results in an estimated 5,000 deaths (Lassa Fever CDC, 2015). These cases are primarily localized in the Western African nations of Sierra Leon, Liberia, Guinea, and Nigeria. In South America, JUNV, MACV, GTOV, and Sabia cause fewer total numbers of disease cases but human infections result in higher case fatality rates. According to the most recent reports AHF, BHF, and VHF result in 15–20, 35, and 33% case fatality rates, respectively (de Manzione et al., 1998; Enria et al., 2004; Patterson et al., 2014).

Lassa virus and JUNV cause the prototypic human disease manifestations for the Old World and New World viruses, respectively. Lassa fever is a zoonotic disease usually resulting from contact with infected murine feces or urine, however, human-to-human transmission is possible. Lassa virus infections have an average incubation time of 10 days and are usually characterized by general flu-like symptoms: fever, malaise, and headache. Patients that progress to severe cases of LF can then develop hemorrhaging and/or neurologic involvement that may be fatal (Frame et al., 1970; Monath et al., 1974; Monson et al., 1984, 1987; McCormick et al., 1987; Hirabayashi et al., 1988; Frame, 1989; Bausch et al., 2001; Enria et al., 2004). In LF survivors, high rates of neurological sequelae have been observed, with nearly 25% of survivors developing deafness (Liao et al., 1992; WHO, 2005; Okokhere et al., 2009). JUNV infection also occurs from exposure to infected murine feces and urine, mostly associated with agricultural activities, and may develop into AHF with an incubation time of 6–14 days. Like LF, AHF initially presents with flu-like symptoms including fever, malaise, anorexia, and headache. Disease progression can result in neurologic symptoms, leukopenia, and thrombocytopenia. One hallmark of AHF as well as other New World virus infections is the large spike of interferon production during infection that is not seen in Old World virus infections (Enria et al., 2004).

## Viral Life Cycle

### Virus Entry

Virus particles initially enter the host through inhalation of aerosolized particles. It is hypothesized that alveolar macrophages are the first cell types infected due to the route of infection and virus present in macrophages early in infection (Gonzalez et al., 1980). While arenaviruses and antigen have been detected in the pulmonary epithelium of infected humans and animals, it is unclear whether these cells are the initial targets (Walker et al., 1982; Hall et al., 1996). These initial infected macrophages can then move to the draining lymph node, resulting in migration and spread of the virus to various tissues (Gonzalez et al., 1980; Buchmeier et al., 2013). Aside from the different cellular receptors, Old World and New World viruses also utilize different entry pathways into the cell. The process of entry is initiated when the GP1 attaches to the corresponding cellular receptor Old World viruses, such as LASV and LCMV as well as Clade C New World viruses utilize α-dystroglycan, an extracellular matrix protein commonly found in the basement membrane (Cao et al., 1998; Spiropoulou et al., 2002), for entry. LUJV was recently discovered to utilize the neurophin (NRP)-2 receptor to mediate entry (Raaben et al., 2017). Recently, TAM family, C-type lectins, and AxI have also been implicated as candidate receptors for LASV and LCMV (Shimojima and Kawaoka, 2012; Shimojima et al., 2012; Fedeli et al., 2017). This raises the possibility of multiple receptors and entry methods for Old World arenaviruses. For Clade B New World arenaviruses, GP1 attaches to transferrin receptor-1 (Radoshitzky et al., 2007; Helguera et al., 2012). TIM family proteins have also been shown to enhance New World arenavirus infectivity (Jemielity et al., 2013). Additionally, siRNA screens indicate that voltage-gated calcium channel (VGCC) subunits play a role in JUNV cell fusion and cellular entry (Lavanya et al., 2013), indicating possible co-receptors used in New World arenavirus entry. To date, the cellular receptor utilized by Clade A New World arenaviruses has yet to be established. After GP1 attaches to the cellular receptor, the virion is endocytosed. New World viruses, such as JUNV, are endocytosed through a clatherin-dependent pathway, whereas the Old World viruses proceed through an unknown clatherin-independent pathway (Rojek et al., 2008b; Pasqual et al., 2011). This entry indicates the role of Endosomal Sorting Complex Required for Transport (ESCRT) proteins for successful cellular entry (Pasqual et al., 2011). Recent studies additionally indicate a possible role of macropinocytosis pathways in Old World viral entry (Oppliger et al., 2016) (see **Table 1** for summary of arenavirus entry). Following endocytosis and acidification of the endosome, GP2, a class I fusion protein, facilitates membrane fusion and the release of the viral genome and replication complexes into the cell (Eschli et al., 2006). Additional evidence has shown that in the process of LASV entry into the cell acidification of the endosome results in an altered G1 receptor binding domain. This results in GP1 binding Lysosomal-associated membrane protein 1 (LAMP1) and potentially assisting in the fusion activity of GP2 (Cohen-Dvashi et al., 2016). LUJV has been shown to utilize the same strategy but binding to CD63 instead of LAMP1 to mediate fusion and escape from the endosome (Raaben et al., 2017).

**Table 1:** Arenavirus receptor usage and cellular entry.

Virus/group	Major cellular receptor	Entry pathway
**Old world**		
LASV, LCMV	α-dystroglycan (Cao et al., 1998)	Clatherin-independent (Rojek et al., 2008b; Pasqual et al., 2011)
LUJV	NRP-2 (Raaben et al., 2017)	Unknown
**New world**		
Clade A viruses	Unknown	Unknown
Clade B viruses	Transferrin receptor-1 (Radoshitzky et al., 2007; Helguera et al., 2012)	Clatherin-dependent (Rojek et al., 2008b)
Clade C viruses	α-Dystroglycan (Spiropoulou et al., 2002)	Unknown

*LASV, Lassa virus; LCMV, Lymphocytic choriomeningitis virus; LUJV, Lujo virus.*

### Replication

**FIGURE 1:**
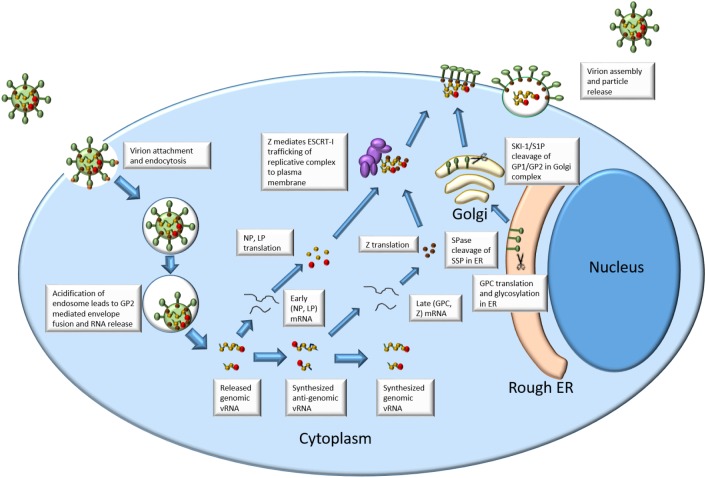
Arenavirus life cycle.

See **Figure 1** for an outline of arenavirus replication.

Once the replication complex has been released into the cytoplasm, the viral LP, in conjunction with NP, begins replication of the viral genome. The association of LP, NP, and the viral genome in the cell is considered to be the minimum requirement for initiation of replication (Lee et al., 2000; López et al., 2001; Hass et al., 2004). The LP contains 4 proposed domains (Brunotte et al., 2011). Domain 1 contains the endonuclease domain hypothesized to facilitate acquisition of 5′ mRNA caps critical to transcription during the replication cycle. The 5′ mRNA cap is an m^7^G structure that is found on the 5′ end of eukaryotic mRNA. This cap serves the purposes enhancing mRNA translation and stability (Both et al., 1975; Furuichi et al., 1977). This LP function is due to the domain’s sequence and structure homology with influenza cap-acquisition protein PA as well as enzymatic studies utilizing LCMV Domain 1 expression (Morin et al., 2010). Domain 3 of the LP contains conserved sequences identified as elements of an RdRp (Vieth et al., 2004). Domains 2 and 4 have as yet unknown functions that may relate to stability, regulation, or other protein-protein interactions (Brunotte et al., 2011). Replication is initiated by LP and NP acquiring 5′ caps from host mRNA. These caps prime the initiation of early gene transcription: NP and LP ORFs on the S and L segments, respectively. Since the arenavirus RNA is negative sense, it is unable to be directly translated in its genomic form. This necessitates nascent RNA-dependent RNA transcription that is produced 5′–3′. The NP and LP ORFs are found in this first transcribed region leading to their initial production as early products (Meyer et al., 2002). Transcription is terminated through the utilization of intergenic hairpin structures found in both the L and S segment IGR (Pinschewer et al., 2005). Translated NP binds to viral RNA to facilitate further transcription, replication, and eventually assembly of progeny virus. Crystal structure analysis of NP has shown that the N terminal domain may accommodate the “stolen” host mRNA cap facilitating further transcription (Qi et al., 2010).

The transition from early gene products to late gene products is facilitated through the production of anti-genomic RNA (Meyer et al., 2002). This is produced when the polymerase reads through the IGR hairpins and makes a complimentary RNA genome. These anti-genomic RNAs are then utilized as the template for transcribing mRNAs from the Z and GPC ORFs. Since they require the production of antigenomic RNA and later genomic mRNA (Meyer et al., 2002), Z and GPC constitute the late gene products in the viral replication cycle. GPC is translated in the endoplasmic reticulum where it undergoes N-linked glycosylation and cleavage of the SSP from GP1/GP2 by SPase (Eichler et al., 2003). The majority of type 1 fusion proteins are cleaved by the host furin like proteases, but arenavirus GPC utilizes a divergent pathway (Harrison, 2015). Precleaved GPC is additionally cleaved between GP1 and GP2 by Subtilisin Kexin Isozyme 1/Site 1 Protease (SKI-1/S1P) into the final SSP, GP1, and GP2 in the trans-Golgi network (Lenz et al., 2001; Rojek et al., 2008a). These three cleaved products stay associated and form a tripartite glycoprotein spike in the cellular plasma membrane. This again is unique in the fact that the signal peptide is incorporated into the final virion instead of being degraded following final protein localization. The Z protein is a short peptide from 90 to 99 aa in length depending on viral species (Capul et al., 2011). This protein contains three domains that contribute to its multifunctional nature. The N-terminal domain contains a myristylation site that allows the protein to localize and anchor itself to the plasma membrane (Perez et al., 2004). The central domain contains a Really Interesting New Gene (RING) domain that chelates Zinc ions. This domain has been shown to be critical for Z-NP and Z-LP interaction but not for Z-GPC interaction (Wilda et al., 2008; Capul et al., 2011; Loureiro et al., 2011). Finally, the C-terminal domain of Z contains proline-rich motifs known to interact with the ESCRT machinery. This interaction is due to interactions of the late-domain of Z with the ESCRT proteins (Perez et al., 2003). Within arenaviruses, there are many different late domains that fall into several large groups. In general, Old World viruses contain the PTAP and PPPY amino acid domains whereas the New World viruses generally contain the PT/SAP amino acid domain (Urata and de la Torre, 2011).

### Assembly and Budding

Interaction with the ESCRT machinery allows for transport of Z to the membrane to support virion budding. Specifically, the late domain of Z has been show to interact with Tumor susceptibility gene 101 (Tsg-101) of the ESCRT pathway (Wilda et al., 2008). Recently, it has been suggested that the late domain motif of LCMV drives the production of defective interfering particles (Ziegler et al., 2016). This is thought to be mediated by the PPPY late domain found in some Old World viruses but not in New World (Ziegler et al., 2016). Accumulation of Z protein performs many late-replication functions in the cell. First, the Z protein interacts with LP and inhibits polymerase function (Kranzusch and Whelan, 2011). In TACV, the LP-Z interaction has been mapped to residues in Domain 1 and Domain 3 of the L polymerase (Perez et al., 2003). This interaction, along with NP-Z interactions, facilitates translocation of the replication complex to the cell membrane in preparation for virion budding that occurs through the interaction of the Z late domain with Tsg-101. Finally, accumulation of the Z protein at the membrane results in viral budding and the production of new, infectious virions. In the absence of other viral proteins and genomic RNA, Z has been shown to initiate self-budding resulting in production of virus-like particles which is why it is considered to be the minimal budding factor for the virus (Hoenen et al., 2007; Ziegler et al., 2016).

## Immune Evasion

During replication, viral evasion from the immune system is critical for productive replication and dissemination in the host. Arenaviruses have been shown to accomplish this in many ways. First, the NP protein has been shown to have strong anti-immune functions in the cell. Interferon regulatory factor 3 (IRF-3) is an important component of the type 1 interferon response. Studies have shown that NP inhibits IRF-3 activation of downstream genes resulting in a limited interferon response (Martínez-Sobrido et al., 2007, 2008). Interestingly, TCRV does not exhibit this same function in the NP perhaps suggesting differential adaptation to an alternative host. The downregulation of interferon-beta in infected cells has been linked to the exonuclease function of the C terminal domain of NP (Qi et al., 2010). This results in the degradation of small dsRNAs that may trigger host signal peptides. One of these potent cellular defenses against viral infection is the dsRNA activated protein kinase (PKR). PKR is able to recognize dsRNA in the cell and become phosphorylated (García et al., 2006). Phosphorylated PKR triggers phosphorylation of other signal proteins as well as the phosphorylation of transcription factors needed to halt viral replication. While some studies have shown that the NP of JUNV is able to stop phosphorylated PKR from resulting in downstream signally and phosphorylation of translation factor eIF2α (King et al., 2017) others indicate that JUNV and MACV infections still produce phosphorylated eIF2α (Huang et al., 2017). Recent studies have demonstrated that while JUNV and MACV activate PKR, LASV infection does not (Huang et al., 2017). It was also shown that this was not due to a prevention of PKR activation, but likely an avoidance of detection. In this way LASV may avoid activation of immune responses in infected cells.

In New World viruses, the Z protein has been shown to also play a role in immune evasion. The Z protein binds to the retinoic acid-inducible gene I (RIG-I) and inhibits interferon-beta production (Fan et al., 2010). RIG-I normally acts as a cellular sensor for double stranded RNA and activates interferon production through a signal cascade (Loo and Gale, 2011). The binding with New World viral Z prevents RIG-I from binding mitochondrial antiviral signaling protein (MAVS), the next protein in the signaling cascade. This halts interferon-beta production and allows the virus to replicate without the presence of a robust immune response (see **Table 2** for a summary of arenavirus immune evasion).

**Table 2:** Arenavirus immune evasion.

Viral protein	Mechanism of immune evasion
NP	Decreased Type I IFN through inhibition of IRF-3 activation (except TACV) (Martínez-Sobrido et al., 2007, 2008)
	Decreased IFN-β production through inhibition or avoidance of PKR signal cascade (Huang et al., 2017; King et al., 2017)
New world Z	Decreased IFN-β production through inhibition of RIG-I signal cascade (Fan et al., 2010)

Another way in which arenaviruses may activate the innate immune system and promote a Type I interferon response is through the activation of various Toll-like receptors (TLRs) (Vidya et al., 2018). JUNV infection has been shown to induce a Type I response mediated through activation of TLR 2 (Cuevas et al., 2011; Cuevas and Ross, 2014). However, induction of this response did not result in decreased viral replication in brain cells. Old World LCMV has been shown to produce a TLR 7 and TLR 9 mediated Type I response (Lee et al., 2009; Macal et al., 2012; Walsh et al., 2012; Buechler et al., 2015). A productive TLR 7 mediated response was also shown to be necessary for robust adaptive immune responses as well as monocyte recruitment (Macal et al., 2012; Walsh et al., 2012). Interesting, chronic LCMV infection of dendritic cells inhibited the TLR 7 and 9 responses of cells treated with each respective agonist (Cuevas and Ross, 2014) in an *ex vivo* model. When these agonist were used to treat *in vitro* cells chronically infected with LCMV, there was no significant difference in the TLR 7 and 9 response. Further studies are necessary to determine the exact contributions of each of these signaling pathways and the viral impact on their activation.

Finally, Old World and New World viruses appear to exhibit differences in terms of immune protection against fatal infection. In New World JUNV, passive antibody transfer from immune sera has been shown to dramatically improve survival of infection in humans (Maiztegui et al., 1979; Ruggiero et al., 1986). Old World LASV, however, does not show any improved survival upon treatment with immune sera (McCormick et al., 1986). It is important to note that some patients treated with the JUNV immune plasma did develop late neurological complications. These results indicate an importance of a B cell mediated response in JUNV infection and a likely T cell mediated response in LASV infection.

## Conclusion

The family *Arenaviridae* encompasses a large variety of viral species found in a variety of animal hosts. Within the genus *Mammarenavirus*, great diversity is still seen in terms of cellular entry strategies, and host immune evasion. Old World and New World viruses demonstrate a marked divergence in these pathways that makes an understanding of the varied mechanisms crucial to the study of their replication and pathogenesis. Within these broader groups there is still an observed divergence in cellular receptor usage and natural host range adding additional complexity to their life cycles. As arenavirus research continues, similarities and differences between the varied species will further allow for the development of pathogenesis models and treatments that are encompassing for species that share those similarities, but not be mistakenly utilized on divergent members. This refinement of arenavirus biology is crucial for all future research and development utilizing these agents.

## Author Contributions

SH compiled and wrote the manuscript. TK, JM, and SP were involved with reviewing and editing.

## Conflict of Interest Statement

The authors declare that the research was conducted in the absence of any commercial or financial relationships that could be construed as a potential conflict of interest.
